# Evaluating a Rapid Immunity Test to Predict Dairy Calf Mortality Risk

**DOI:** 10.3390/biology14060584

**Published:** 2025-05-22

**Authors:** Ansley M. Roper, Caroline Guzi Savegnago, Thiago N. Marins, Jing Gao, Rui Xie, Sha Tao, Qun Huo

**Affiliations:** 1 Department of Animal and Dairy Science, University of Georgia, Athens, GA 30602, USA; aroper2@utk.edu (A.M.R.); carolguzi@uga.edu (C.G.S.); tnmarins@uga.edu (T.N.M.); jing.gao@uga.edu (J.G.); 2 Department of Statistics and Data Science, University of Central Florida, Orlando, FL 32816, USA; rui.xie@ucf.edu; 3 Department of Chemistry and Nanoscience Technology Center, University of Central Florida, Orlando, FL 32826, USA

**Keywords:** humoral immunity, D2Dx immunity test, calf mortality risk, transfer of passive immunity

## Abstract

The study objective was to compare the ability of Brix values and the D2Dx immunity test to predict the mortality risk of calves under 30 days of age. Heifer calves at 2–3 days of age were enrolled from two farms: Farm A, which has a low calf mortality rate (n = 849, 2.9% mortality), and Farm B, which has a high calf mortality rate (n = 698, 11.7% mortality). Serum samples were collected from the calves when they were 2–3 days of age. The D2Dx scores and Brix values of serum samples were compared between calves that died or survived between 3–30 days of age. For both farms, calves that survived their first 30 days of life had greater serum Brix values (9.05 vs. 8.48, *p* = 0.002 for Farm A; 9.26 vs. 8.99, *p* = 0.05 for Farm B) and D2Dx scores (0.0281 vs. 0.0036, *p* < 0.001 for Farm A; 0.0342 vs. 0.0286, *p* = 0.05 for Farm B) at 2–3 days of age compared with calves that died. On Farm A, the D2Dx score shows significantly better sensitivity (96% vs. 48%) and Area Under the Curve (0.87 vs. 0.69) than the Brix value in predicting calf mortality. However, similar results were not observed for the high-mortality farm (Farm B) due to other factors in addition to calf immune status that influenced calf survival. This study concludes that the D2Dx immunity test can be used to identify newborn dairy calves with low immunity and increased mortality risk; however, the prediction is most effective if no other risk factors are involved.

## Simple Summary

Transfer of passive immunity (TPI) is critical for the survival of pre-weaned dairy calves. Plasma or serum Brix values measured by a refractometer are often used in farm settings to estimate circulating immunoglobulin (Ig) G concentration as a proxy for TPI. However, the Brix value only estimates the total solid percentage in blood and does not directly reflect the immune status of the calf. The D2Dx (from diameter to diagnostics) immunity test is a new nanoparticle-enabled rapid blood test to measure humoral immunity in humans and animals. In this study, we compared the performance of Brix value and the D2Dx immunity test in predicting neonatal dairy calf mortality risk. Our study found that the D2Dx immunity test outperforms Brix value when predicting neonatal diary calf mortality risk within their first 30 days of life if no other risk factors are involved. The D2Dx immunity test can potentially be used as a new tool in farm settings for dairy calf health management.

## 1. Introduction

The United States has a total of about 9 million dairy cows, and worldwide, there are more than 270 million dairy cows. The dairy industry and farms represent a major portion of the global economy. The calf growth performance determines the financial success of dairy farms, and it is paramount to raise healthy replacement heifers. At birth, the immune system of a calf is immature and unprimed, exposing the calves to a greater risk of infectious diseases. In the United States, depending on the size of the dairy farm, 5.7 to 10.5% mortality rates have been reported for calves prior to weaning [1]. Newborn calves can obtain the required immune protection by consuming sufficient high-quality colostrum immediately after birth [2,3], a process called transfer of passive immunity (TPI). Colostrum is the first milk collected from the dam following parturition and contains numerous nutrients and immune components. The intestinal epithelium of a newborn calf is permeable and allows non-selective transport of intact and large immune components into the neonatal circulation [3,4]. These immune components include those involved in the acquired immune system, such as immunoglobulins (Ig) and lymphocytes, as well as those involved in innate immunity, such as neutrophils, macrophages, complements, acute phase proteins, various saccharide polymers, and antimicrobial proteins [5,6]).

The quantification of TPI is critical for identifying animals with high risk of disease and mortality. Immunoglobulin G, particularly IgG1, is the most abundant Ig in colostrum and the major Ig transferred into the neonatal circulation. Thus, IgG is the most studied immune component to assess colostrum quality and TPI. TPI can be categorized into four categories based on serum IgG concentration: excellent, with serum containing at least 25 g/L of IgG; good (18.0–24.9 g/L); fair (10.0–17.9 g/L); and poor (less than 10 g/L [7]. In the United States, the percentage of dairy heifers with excellent, good, fair, and poor TPI is 35.5, 25.7, 26.8, and 12%, respectively [7]. However, direct measurement of IgG levels in calf serum is not practical on farms [8]. Instead, the IgG level is often estimated using a digital Brix refractometer [9,10]. Brix refractometers measure the total solid content in a blood serum sample, which includes but is not limited to IgG. The corresponding Brix values for excellent, good, fair, and poor TPI are >9.4, 8.9–9.3, 8.1–8.8, and <8.1%, respectively [7].

It is important to note that TPI is the transfer of an array of immune components, not only Ig. Measurements such as IgG and Brix are proxies for evaluating the transfer of immune components following colostrum ingestion. To our best knowledge, we have not seen clear evidence from previous studies that Brix value can be used to predict calf mortality risk. New technologies and tests that can measure immune status more accurately in farm settings and, hence, predict the potential health outcomes of neonatal animals can be very valuable in livestock management.

The D2Dx immunity test is a new nanotechnology-enabled immune response test to measure humoral immunity in humans and animals [11,12]. The D2Dx immunity test uses a gold nanoparticle as a pseudo pathogen to probe the immune response capability of a serum or plasma sample [11,12]. Upon mixing the gold nanoparticle reagent solution with the sample, humoral immune molecules will react with the nanoparticle probe, causing nanoparticles to agglutinate together and change color. In our previous publication [11], we demonstrated that the three most important humoral immune molecular components—IgG, IgM, and complement proteins—are collectively involved in the reaction with the nanoparticle probe. Eliminating these molecules from blood serum or adding them back to it can change the test score dramatically. The nanoparticle–immune molecule interaction mimics the immune response in vivo, where pathogens such as viruses are agglutinated together into large clusters through binding and recognition with both specific and non-specific IgG, IgM, and complement proteins [13,14]. By replacing real pathogens with a gold nanoparticle probe, the nanoparticle aggregate formation is detected by monitoring the color change of the nanoparticles, a property known as surface plasmon resonance shift [15]. The test score represents the immune reactivity of a sample rather than the quantity of individual immune molecules.

The D2Dx immunity test involves a single-step process with results obtained in less than 1 min. It can be readily conducted in farm settings. A recent study conducted on a calf ranch found a positive correlation (r = 0.61, *p* < 0.01) between the D2Dx immunity test scores and total serum protein measurement of newborn Holstein bull calves [16], suggesting the potential of the D2Dx immunity test for evaluating TPI. Because the D2Dx test measures the actual humoral immune response of an animal, we hypothesize that the blood D2Dx test score at 2–3 days of age may be a better indicator of calf mortality risk compared with Brix values. The study objective was to compare the ability of Brix values and D2Dx test scores to measure the immune status of dairy calves following TPI and to predict the mortality risk of calves within one month of age. Knowing the true immune status of neonatal calves will help farmers find more effective immune modulation products and management solutions to reduce calf mortality and financial losses.

## 2. Materials and Methods

### 2.1. Animals

**Figure 1:**
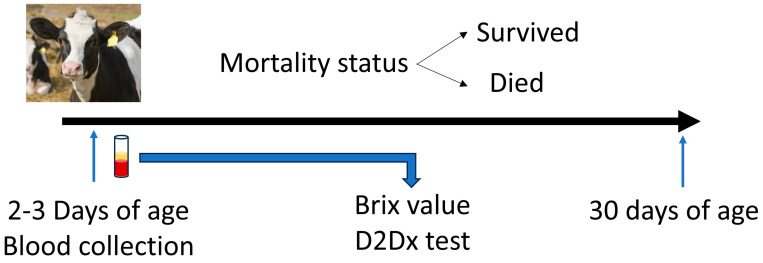
The experimental design of the study.

This study was conducted on two commercial dairy farms located in Georgia. All experimental and animal handling procedures were approved by the University of Georgia Institutional Animal Care and Use Committee (AUP#: A2022 09_015_Y1_A0). The design of the study is outlined in Figure 1.

On Farm A, immediately after birth, newborn Holstein calves were vaccinated with a trans nasal respiratory vaccination (Inforce 3, Zoetis, Parsippany, NJ, USA), 5 mL Vitamin E-AD (VetOne, MWI Animal Health, Boise, ID, USA) was administered, and their navels were dipped with iodine (iodine tincture 7%, Thatcher Group, Salt Lake City, UT, USA). Within 2 h of birth, calves were fed 1.9 L of high-quality colostrum (Brix value ≥ 22.5%) pasteurized using the Perfect Udder Bag Pasteurizer (Dairy Tech Inc., Greely, CO, USA). Following colostrum feeding, calves were moved into individual polyethene hutches bedded with sand where they were raised until weaning. The hutches were in an open area away from the main herd, without shade. Calves were fed 4.5 L pasteurized waste milk equally divided into two feedings per day (0700 and 1400 h) until weaning, which occurred at 64 days of age after feeding calves once a day (2.25 L, 0700 h) for 7 d. Water and calf starter grain were provided ad libitum starting from 2 days of age.

On Farm B, each Holstein calf was administered with a trans nasal respiratory vaccination (Inforce 3, Zoetis, Parsippany, NJ, USA) and their navels were dipped with Triodine-7 (Priority Care, First Priority Inc., Elgin, IL, USA) immediately after birth, and they were then transferred into a small trailer and fed 3.8 L unpasteurized colostrum (Brix ≥ 22%). Calves were transported from the trailer to an enclosed barn once a day and housed in individual wire pens bedded with straw. Fans were provided in the barn for cooling and turned on from 1000 h to 2000 h during summer. Starting at 2 days of age, calves were fed a milk replacer containing 20% protein and 20% fat (as fed basis) medicated with lasalocid (79 mg/kg) (Southeast Milk, Inc., Mayo, FL, USA) twice daily at 0600 and 1700 h. Calves were fed 1.9 L per feeding for one week then transitioned to 2.8 L per feeding via buckets for 53 days. At 61 days of age, calves were fed once per day (2.8 L) at 0600 h for one week until they were weaned. Calf starter grain (22% CP, Ampli-Calf, Purina) was provided ad libitum from 2 days of age and water was provided ad libitum from 21 days of age. Electrolytes (BlueLite C, TechMix, Stewart, MN, USA) were offered to all calves between morning and afternoon feedings. Calves remained in the barn for one more week post weaning.

### 2.2. Sample and Data Collection

From November 2022 to January 2024, all living heifer calves at 2 days of age were enrolled (Farm A: n = 849; Farm B: n = 698). The birth dates of the enrolled calves ranged from 30 October 2022 to 31 January 2024. At 2–3 days of age, blood was collected through the jugular vein into a vacutainer tube without anti-clotting factors (BD Vacutainer, Becton, Dickinson and Company, NJ, USA). Samples were kept at room temperature for 3–6 h before centrifugation at 3000× *g* at 4 °C for 30 min to collect serum. Samples were stored at −20 °C for future analysis. All calves were monitored for mortality from 3–30 days of age. The passive immunity transferred from their dam’s colostrum to neonatal calves is known to last for about one month (4–5 weeks). Hence, to study the correlation between the transfer of passive immunity and mortality risk, the calf mortality was tracked until up to about 30 days of life.

On Farm A, a total of 25 calves died within 3–30 days of age, resulting in a mortality rate of 2.9% during the first month of life. On Farm B, a total of 82 calves died during the experimental period, resulting in an 11.7% mortality rate from 3 to 30 days of age. On both farms, the causes of death were one or more of following: diarrhea, other digestive diseases (e.g., bloating), respiratory disease, other infections, and unknown. Thus, Farm A is considered a low-mortality risk farm, and Farm B is regarded as a high-mortality risk farm.

### 2.3. Immunity Testing Using Brix or D2Dx Immunity Test

Serum samples collected at 2–3 days of age from the following calves were tested using a Brix refractometer and the D2Dx immunity test: 25 serum samples from calves that died later between 3–30 days of age on Farm A (labeled as “died”); 82 serum samples from calves that died later between 3–30 days of age on Farm B (labeled as “died”); 75 calves that survived between 3–30 days of age on Farm A (labeled as “survived”); and 148 calves that survived between 3–30 days of age on Farm B (labeled as “survived”). The samples in the survival group were randomly selected to match the birthdays of the calves that died between 3–30 days of age. The samples were analyzed as blind samples.

Brix values were obtained using a digital Brix refractometer (MISCO AP201, Misco, Solon, OH, USA) according to the instructions. The D2Dx immunity test was conducted using test kits and a CT-100 colorimeter reader device (Nano Discovery Inc., Orlando, FL, USA). Briefly, to conduct the D2Dx immunity test, 50 µL test reagent was transferred to a mini cuvette. To the reagent solution, 10 µL serum was added. After vortex-mixing for 5 s, the cuvette was placed in the CT-100 device. The absorbance of the mixture solution was measured once immediately and then again 30 s later. The difference between the two absorbance values is the D2Dx immunity score.

### 2.4. Statistical Data Analysis

Data collected from each farm were analyzed separately. The D2Dx scores and Brix values were analyzed using the PROC GLM procedure of SAS 9.4 (SAS Institute Inc., Cary, NC, USA) and the glm function of R 4.2.2 (R Foundation for Statistical Computing, Vienna, Austria). Statistical analyses were conducted to evaluate the associations between the D2Dx score, Brix value, and mortality outcomes (survived vs. died). Descriptive statistics, including box–whisker plots and t-tests, were utilized to explore the distribution and differences between groups. The Shapiro–Wilk normality test was used to test the normality of the distributions of D2Dx scores and Brix values. To assess the relationship between the D2Dx score and the Brix value, a Spearman correlation analysis was performed. Spearman’s rank correlation was chosen because the D2Dx score was not normally distributed, whereas the Brix value followed a normal distribution. Predictive modeling was performed using logistic regression, where mortality (survived vs. died) served as the dependent variable and the serum D2Dx score or Brix value served as the primary predictor. Model performance was assessed using various metrics, including sensitivity (ability to correctly identify death cases), specificity (ability to correctly identify alive cases), positive predictive value (PPV, proportion of predicted death cases that were truly dead), and negative predictive value (NPV, proportion of predicted alive cases that were truly alive). Additionally, the Receiver Operating Characteristic (ROC) Curve and the Area Under the Curve (AUC) were analyzed to summarize the model’s performance across different classification thresholds. The optimal cutoff threshold value for the logistic regression model is identified as the point where the sum of sensitivity and specificity is maximized.

## 3. Results

**Figure 2:**
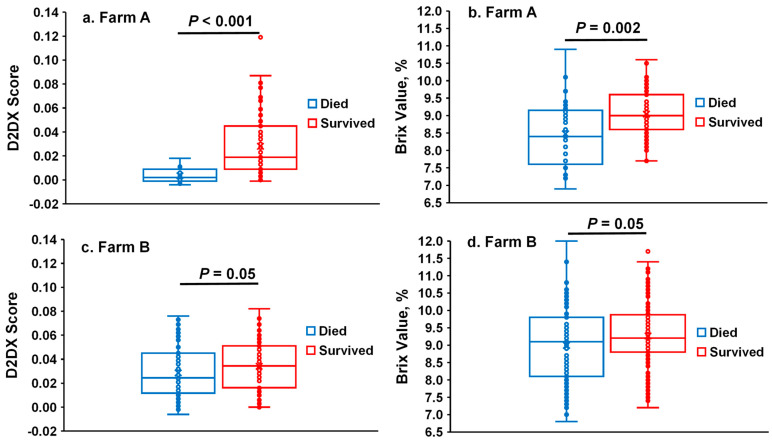
Box and whisker plots of the D2Dx scores and Brix values of serum samples collected from calves that died or survived within 30 days of age on Farm A and Farm B. (**a**,**c**)–D2Dx scores; (**b**,**d**)–Brix values.

The D2Dx scores and Brix values of calf serum samples collected at 2–3 days of age were compared between calves that survived or died later. On Farm A, calves that survived their first 30 days of life had a greater serum Brix value (mean values: 9.05 vs. 8.48, respectively; *p* = 0.002) and D2Dx score (0.0281 vs. 0.0036, respectively; *p* < 0.001) at 2–3 days of age than calves that died at 3–30 days of age. However, as shown in the box–whisker plot (Figure 2a), the D2Dx scores of the calves that died later on Farm A displayed a narrow range of variability while the D2Dx scores of the calves that survived had a wide range of variability. In contrast, the Brix values of both groups of calves had a wide range of variability (Figure 2b). On Farm B, calves that survived up to 30 days of age also had greater serum Brix values (9.26 vs. 8.99, respectively; *p* = 0.05) and D2Dx scores (0.0342 vs. 0.0286, respectively; *p* = 0.05) compared with the calves that died. However, the D2Dx scores and Brix values of both groups of calves exhibit wide ranges of variability (Figure 2c,d). For both farms, regardless of the mortality status, D2Dx scores were significantly and positively associated with Brix values (Farm A: Spearman’s ρ = 0.42, *p* < 0.001; Farm B: Spearman’s ρ = 0.42, *p* < 0.001). This finding is similar to what has been previously reported by Casper [16].

The serum concentrations of IgG and the total protein of calves following colostrum consumption are associated with mortality [17]. Poor TPI, indicated by low total serum protein, is associated with increased mortality risk in pre-weaned dairy calves [18]). Using data collected from the USDA National Animal Health Monitoring System’s Dairy 2014 study, Lombard et al. [7] reported that calves with poor TPI, determined by serum IgG concentration, had a lower survival rate than calves with fair, good, or excellent TPI before 60 d of age (7.4, 3.8, 1.5, 2.5% mortality rate, respectively). Similarly, in our study, we found that on Farm A, when using the serum Brix value as the proxy, calves with poor TPI had a greater mortality rate (69.2%) within the first month of life compared with those with fair, good, or excellent TPI (20.0%, 21.7%, and 13.8%, respectively). It is important to note that the mortality rates reported herein were calculated based on calves enrolled in blood analysis only (n = 100, 25 died and 75 survived); thus, the values are much higher than those reported by Lombard et al. [7]. On Farm B (n = 230, 82 died and 148 survived), the mortality rates of calves with poor or fair TPI (47.4% and 43.9%, respectively) were higher than calves with good or excellent TPI (31.5% and 29.9%, respectively), but the differences in mortality rates between different TPI groups were smaller than those of Farm A.

Calves on farms A and B had comparable Brix values at 2–3 days of age, suggesting that both farms have effective colostrum management. Thus, the greater calf mortality rate of Farm B compared with Farm A is most likely due to nutritional and other management issues. These may include but are not limited to water availability (offered at 2 vs. 21 days of age), liquid feeds (pasteurized waste milk vs. 20:20 milk replacer), bedding management (sand vs. straw), ventilation (outdoor housing vs. indoor housing), and hygiene. Despite the importance of TPI, good management is the key to maintaining a healthy herd. Data from this study strongly suggest that good passive immunity is a prerequisite but cannot guarantee the low mortality of pre-weaned dairy calves.

Although lower serum IgG content and Brix values are associated with increased morbidity and mortality rates, neither value has good sensitivity to mortality. For instance, in the dataset reported by Lombard et al. [7], only 28% of the death events involved calves with poor TPI (=21/75 [# of calves that died with poor TPI/total # of calves that died]). Similarly, in our study, only 36% (9/25) of the death events on Farm A and 22% (18/82) on Farm B involved calves with poor TPI based on serum Brix values, further confirming that Brix value is not effective in predicting calf mortality risk. A test that can evaluate the immune responses of newborn calves and accurately predict calf mortality is urgently needed.

**Figure 3:**
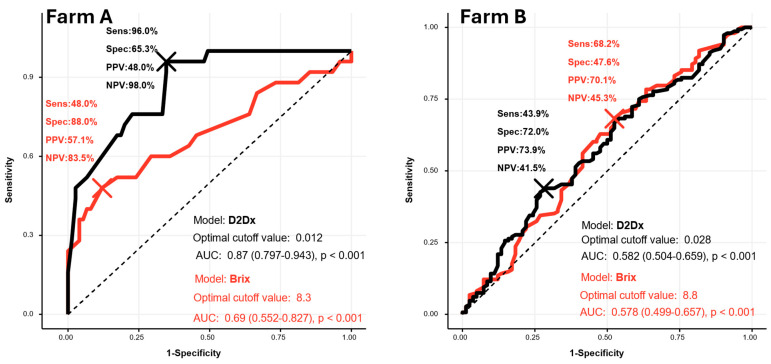
The Receiver Operating Characteristic (ROC) curves and Area Under the Curve (AUC) values of the D2Dx-based and Brix-based predictive models used on farms A and B. The sensitivity (Sens = [true positive]/[true positive + false negative]), specificity (Spec = [true negative]/[true negative + false positive]), positive predictive value (PPV = [true positive]/[true positive + false positive]), and negative predictive value (NPV = [true negative]/[false negative + true negative]) were calculated.

We used logistic regression to predict the mortality outcome (died vs. survived), with D2Dx scores and Brix values as the predictors. To account for the imbalance between the two cases (died or survived), we employed a weighted logistic regression model. We then evaluated the performance of the two logistic regression models by calculating sensitivity, specificity, and positive and negative predictive values for each prediction. Additionally, we assessed the models using Receiver Operating Characteristic (ROC) curves and the Area Under the Curve (AUC), as the ROC curve provides a comprehensive view of the model’s ability to distinguish between the two outcome classes across various probability threshold settings. The probability threshold setting refers to the probability cutoff point at which predicted probabilities are classified into one of the binary outcome categories (e.g., above the threshold predicts “died,” and below it predicts “survived”). On Farm A, as shown in Figure 3, the D2Dx score shows significantly better sensitivity (96% vs. 48%) and Area Under the Curve (0.87 vs. 0.69) than the Brix value when predicting calf mortality. The D2Dx model outperforms the Brix model across all probability threshold settings, providing strong evidence that the D2Dx score is a superior predictor of mortality risk compared with Brix values. However, similar results were not observed in the data collected on Farm B, and the ROC curves for the Brix value and D2Dx score are close to each other. But the sensitivity at the cutoff point of the D2Dx score remains higher than the Brix value, indicating that it is a better predictor of mortality risk (Figure 3).

A post hoc power analysis was conducted for our logistic regression models using the simr package in R, with a Type I error rate of 0.05. For Farm A, the D2Dx test achieved 100% power, indicating a very high likelihood of detecting a true effect if present, while the Brix test reached 89.9% power, demonstrating strong statistical sensitivity. In contrast, for Farm B, the D2Dx test achieved 52.7% power and the Brix test reached 51.5% power, suggesting a lower probability of detecting true effects.

## 4. Discussion

The results of this study suggest that the D2Dx immunity test can be used to evaluate TPI following colostrum ingestion and is an effective predictor of calf mortality risk during the first month of age. In both low- and high-calf mortality rate farms, the D2Dx test outperforms the Brix value in identifying calves with a high mortality risk. However, the prediction is most, or only, effective if no other management-related risk factors are involved. The reason for the discrepancy between the results of low- and high-mortality rate farms may be because, in a high-mortality risk farm such as Farm B, the successful passive immunity of a calf is important, but not a determinant for calf mortality. The management issues present on a high-mortality risk farm dilute the importance of passive immunity for calf survival. In this situation, the success of TPI, or parameters associated with TPI, are not effective predictors of calf mortality.

There are several limitations in our current study: The number of samples analyzed is limited. The ROC curve generated based on data from farm A is not smooth enough, which suggests fluctuations in the model’s performance across different threshold levels. Consequently, the optimal cutoff value identified may not be robust, meaning it could change significantly with a different sample or larger dataset. With a larger sample size, the ROC curve would become smoother, providing more stable and reliable information for determining the optimal cutoff value. In order to refine the cutoff value to predict mortality for farm applications, the study needs to include larger sample sizes, and the samples should be collected from more farms. In this study, only Holstein heifer calves were used for analysis. It is known that gender (male vs. female) and breed have a significant impact on immunity and health. Thus, future studies also need to include male calves and different breeds. Finally, the blood samples we tested were archived frozen serum samples. In actual farm usage, the samples will be tested fresh. Fresh samples may give more accurate D2Dx scores for evaluating immune responses than frozen samples. Thus, future studies to refine the on-farm use of the D2Dx immunity test are urgently needed.

## 5. Conclusions

Productive dairy herds rely heavily on the rearing of healthy calves to provide replacement cows for continuous production. The performance of dairy calves determines the future success of the farm they belong to. Neonatal dairy calves face a high mortality risk due to their immature immune functions upon birth. The successful transfer of passive immunity is essential to protect neonatal calves from infectious diseases during their first 30 days of life. While the Brix refractometer has been recommended as an on-farm tool for monitoring TPI in dairy calves, because Brix refractometers do not directly measure true immune function, the application of Brix refractometers for this purpose appears to be limited. More accurate technologies and tools are needed to measure the true immune status of dairy calves at the neonatal stage. Our study suggests that the D2Dx immunity test could potentially be a new tool to fill this gap. The D2Dx test is easy to conduct and has been applied in farm settings [16] to measure the immune status of newborn calves. Farm owners and veterinarians may use the test to determine an individual calf’s immune health or as a tool to monitor the overall herd immune health. The test results can be used to find the most effective products (feed, nutrition supplements, additives) to improve the immune health of neonatal calves and the best management solutions to mitigate their morbidity and mortality risk.

## Data Availability

The raw data supporting the conclusions of this article will be made available by the authors on request.

## Abbreviations

The following abbreviations are used in this manuscript:

TPI	Transfer of passive immunity
D2Dx	From diameter to diagnostics
